# Supplementary material for: Toxicity and Anti-Inflammatory Activity of Phenolic-Rich Extract from Nopalea cochenillifera (Cactaceae): A Preclinical Study on the Prevention of Inflammatory Bowel Diseases
Source: Plants (Basel). 2023 Jan 29;12(3):594. doi: 10.3390/plants12030594 (PMC9921826; doi:10.3390/plants12030594)
Supplement: Supplementary file 1 [file plants-12-00594-s001.zip › plants-2109924-supplementary.pdf]

plants

Supplementary Material

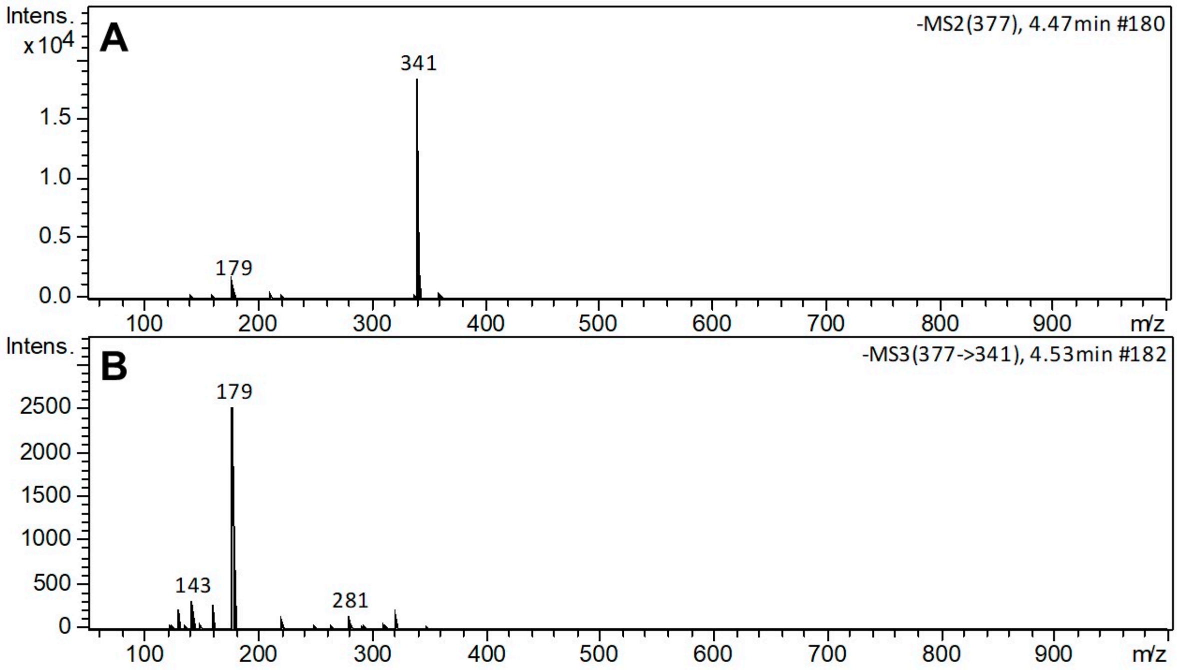

Figure S1. CID-MS^n mass spectra in negative ion mode for compound **1**: (**A**) CID-MS^2 spectrum of the precursor ion at *m*/*z* 377; (**B**) CID-MS^3 mass spectrum of the product ion at *m*/*z* 341.

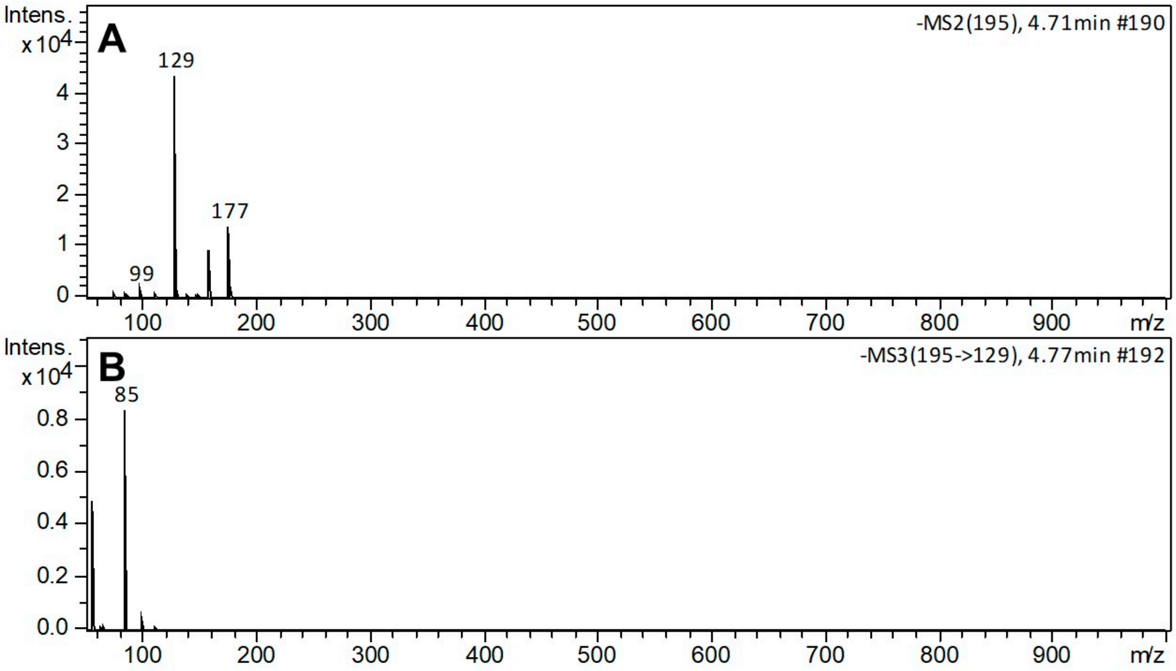

Figure S2. CID-MS^n mass spectra in negative ion mode for compound **2**: (**A**) CID-MS^2 spectrum of the precursor ion at *m*/*z* 195; (**B**) CID-MS^3 mass spectrum of the product ion at *m*/*z* 129.

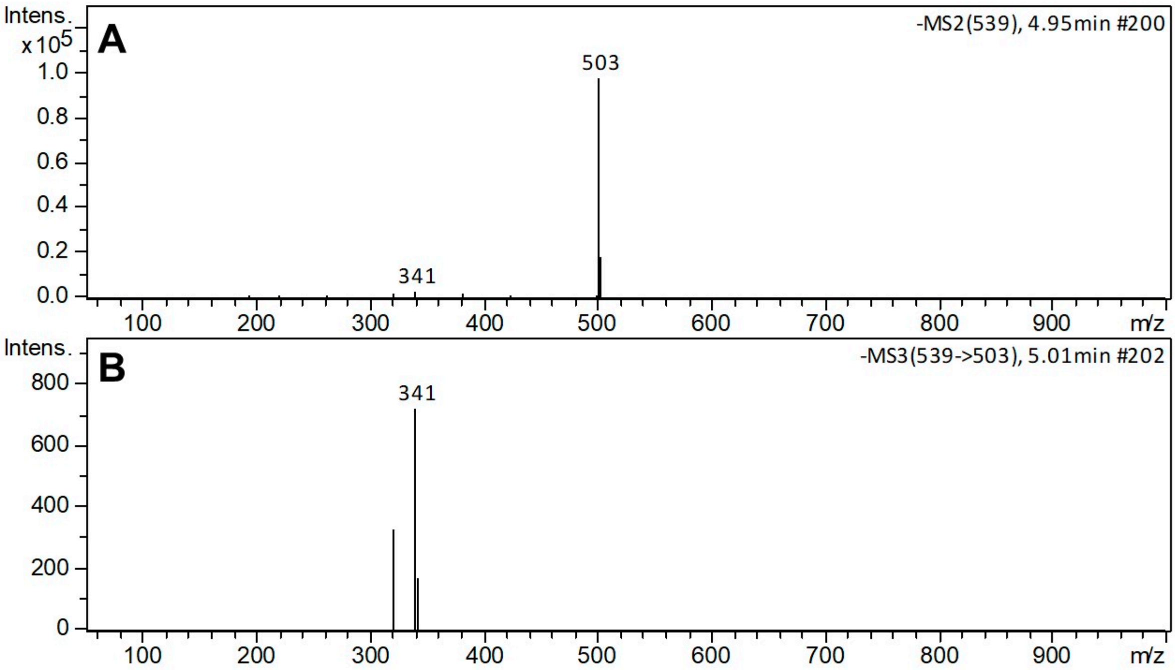

Figure S3. CID-MS^n mass spectra in negative ion mode for compound **3**: (**A**) CID-MS^2 spectrum of the precursor ion at *m*/*z* 539; (**B**) CID-MS^3 mass spectrum of the product ion at *m*/*z* 503.

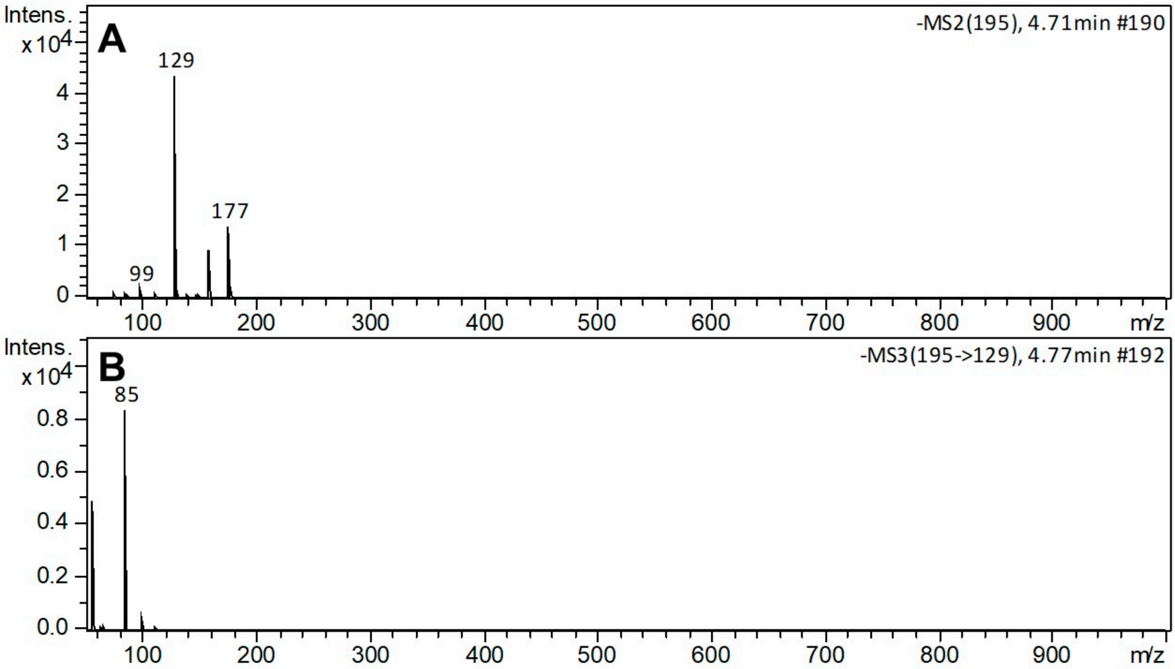

Figure S4. CID-MS^n mass spectra in negative ion mode for compound **1**: (**A**) CID-MS^2 spectrum of the precursor ion at *m*/*z* 377; (**B**) CID-MS^3 mass spectrum of the product ion at *m*/*z* 341.

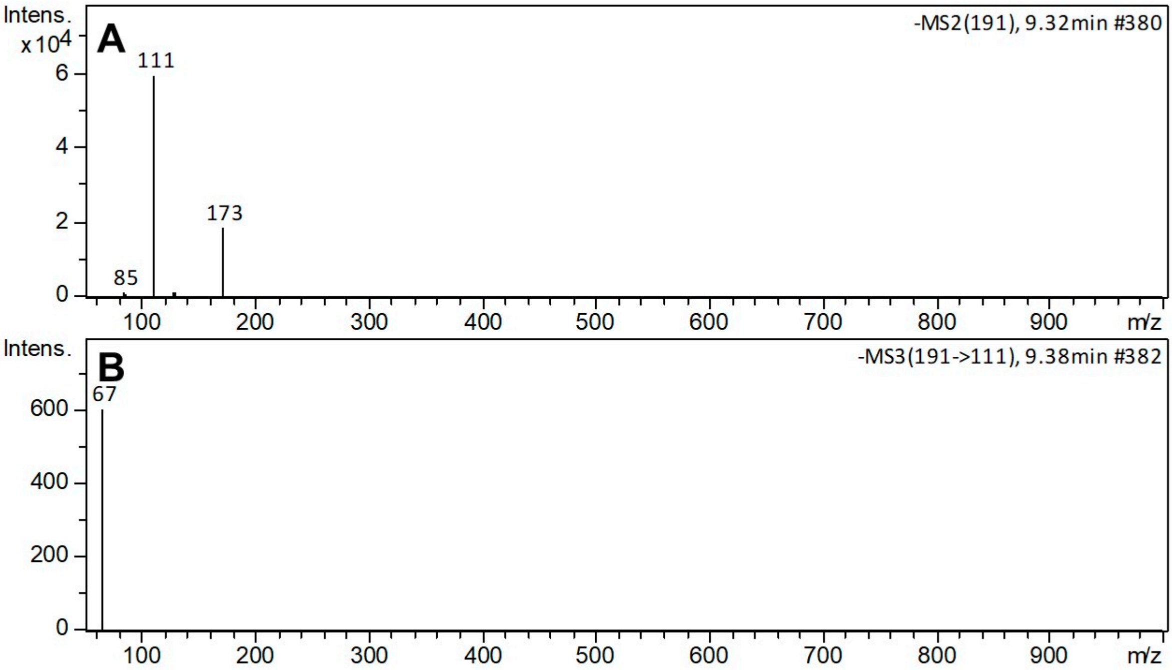

Figure S5. CID-MS^n mass spectra in negative ion mode for compound **5**: (**A**) CID-MS^2 spectrum of the precursor ion at *m/z* 191; (**B**) CID-MS^3 mass spectrum of the product ion at *m/z* 111.

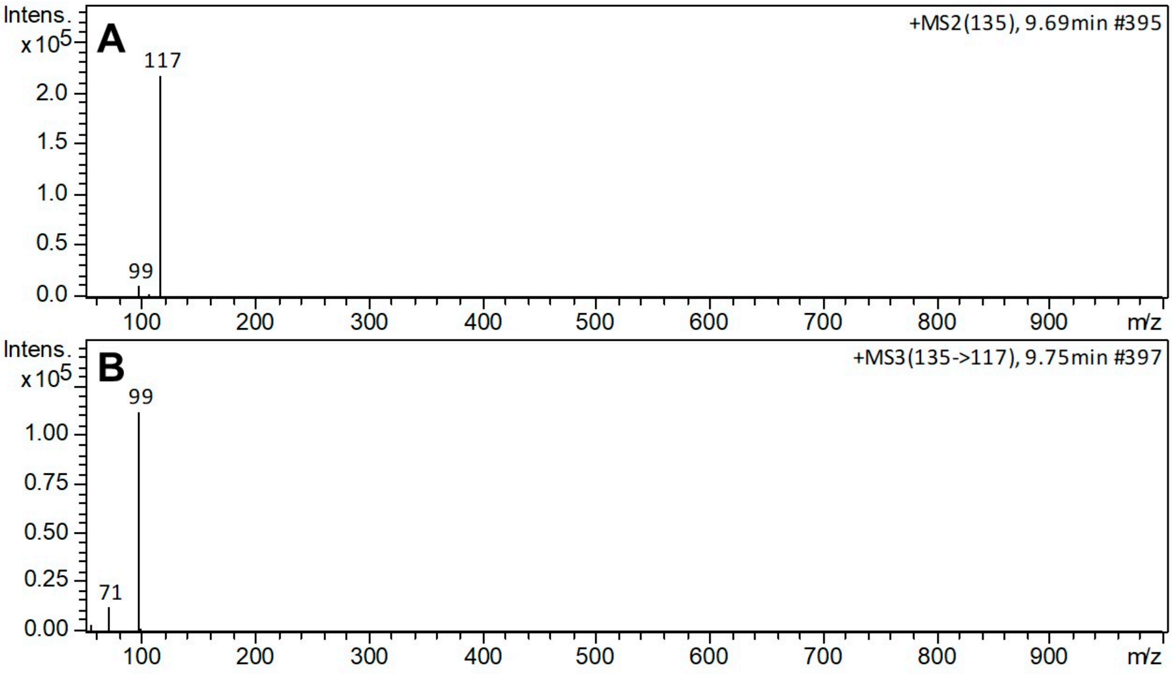

Figure S6. CID-MS^n mass spectra in negative ion mode for compound **6**: (**A**) CID-MS^2 spectrum of the precursor ion at *m/z* 135; (**B**) CID-MS^3 mass spectrum of the product ion at *m/z* 117

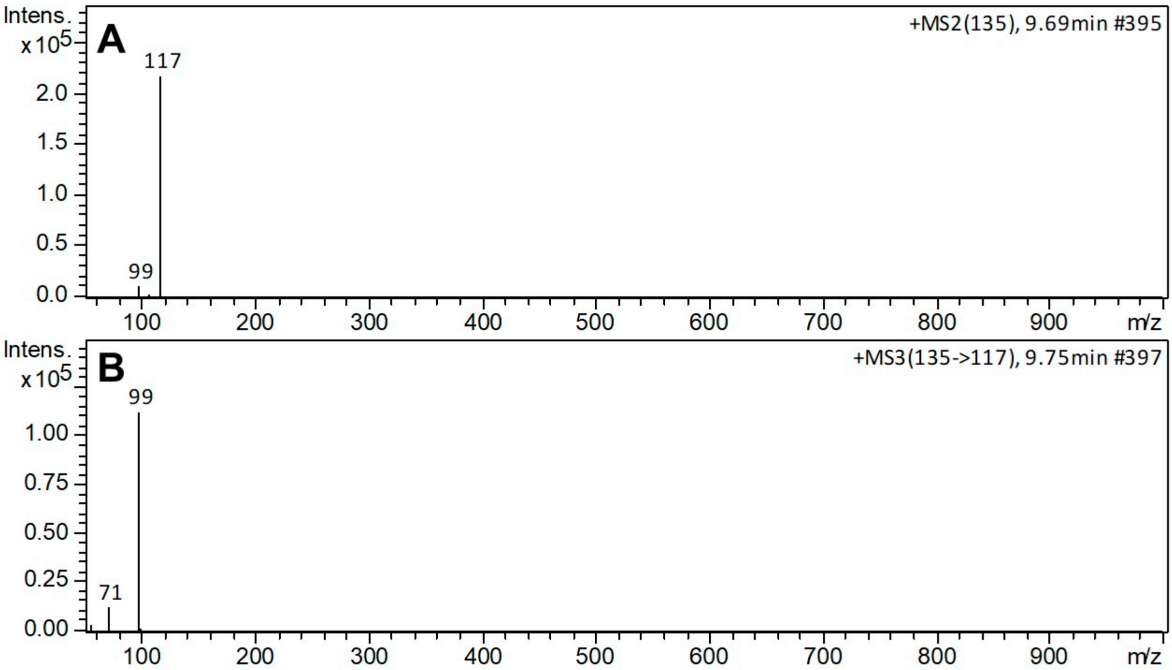

Figure S7. -MS^n mass spectra in positive ion mode for compound **7**: (**A**) CID-MS^2 spectrum of the precursor ion at *m*/*z* 284; (**B**) CID-MS^3 mass spectrum of the product ion at *m*/*z* 152.

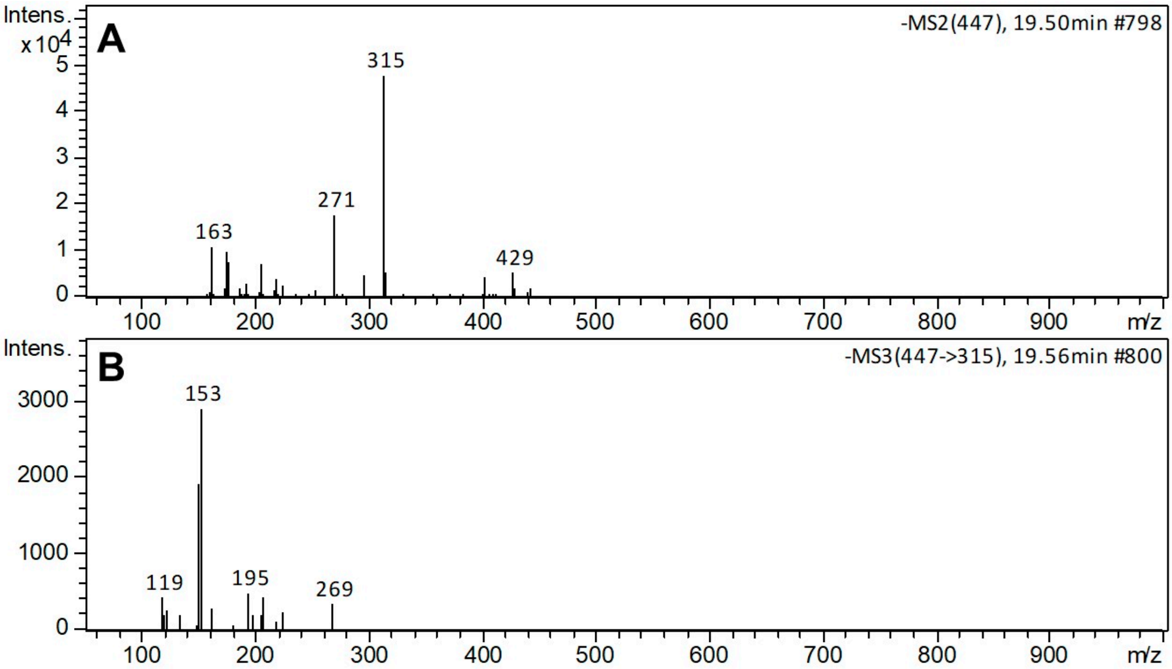

Figure S8. CID-MSn mass spectra in negative ion mode for compound 8: (A) CID-MS2 spectrum of the precursor ion at m/z 447; (B) CID-MS3 mass spectrum of the product ion at m/z 315.

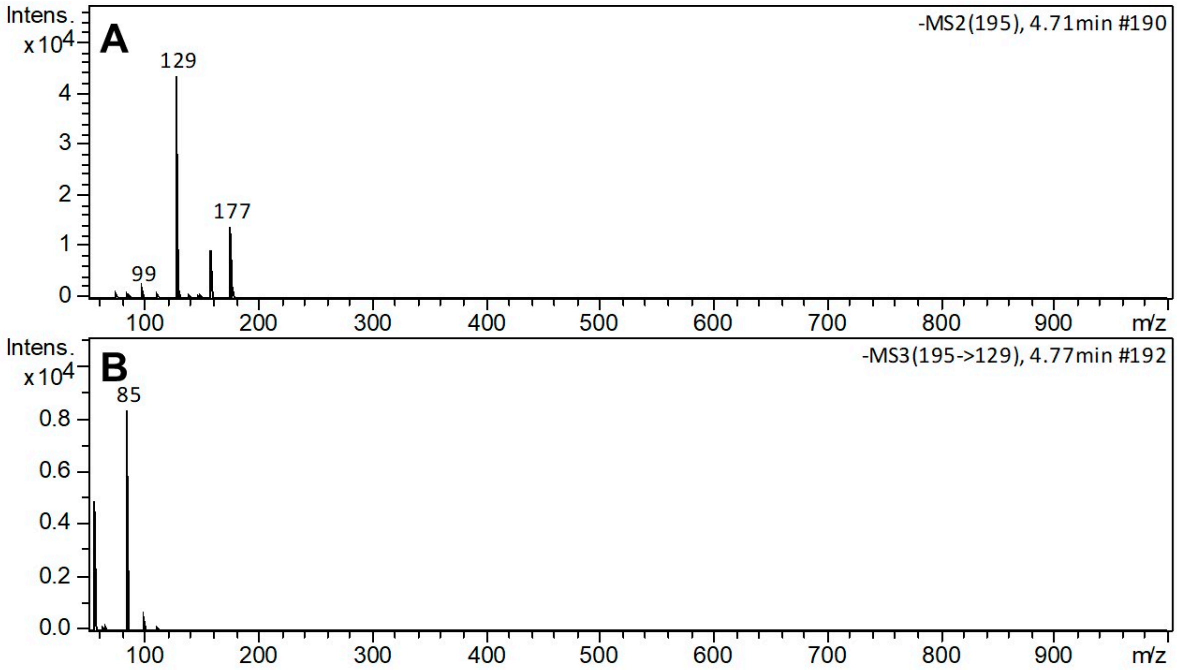

Figure S9. CID-MS^n mass spectra in negative ion mode for compound **9**: (**A**) CID-MS^2 spectrum of the precursor ion at *m*/*z* 447; (**B**) CID-MS^3 mass spectrum of the product ion at *m*/*z* 315.

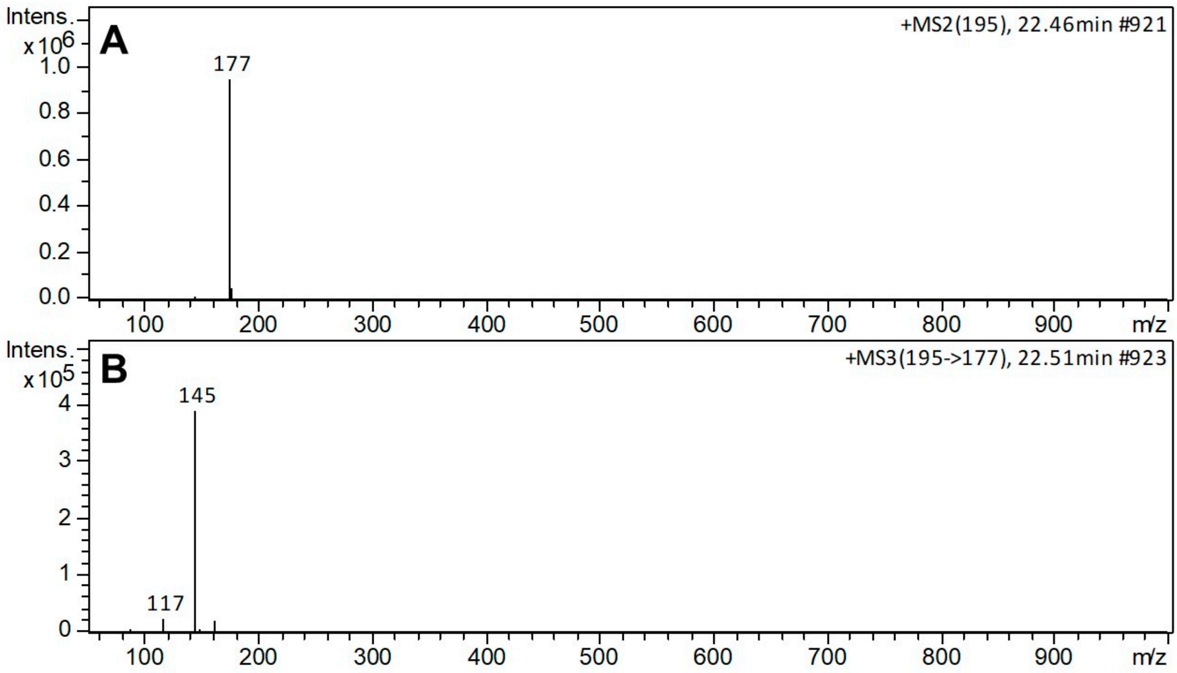

Figure S10. CID-MS^n mass spectra in positive ion mode for compound **10**: (**A**) CID-MS^2 spectrum of the precursor ion at *m*/*z* 195; (**B**) CID-MS^3 mass spectrum of the product ion at *m*/*z* 177.

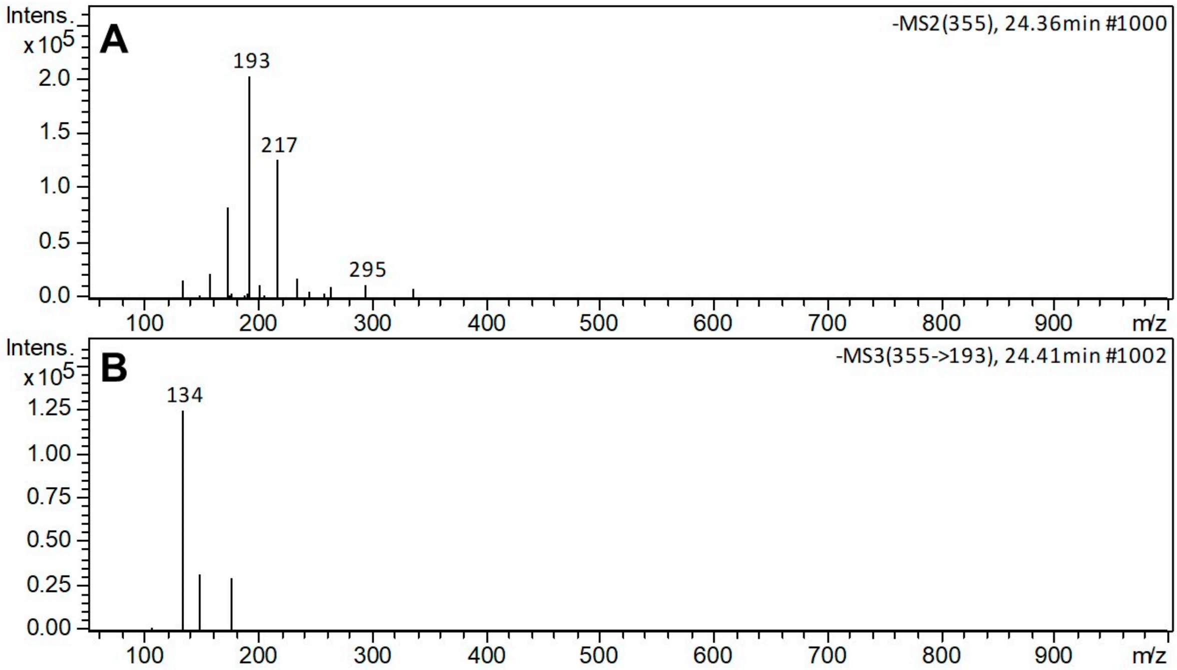

Figure S11. CID-MS^n mass spectra in negative ion mode for compound **11**: (**A**) CID-MS^2 spectrum of the precursor ion at *m*/*z* 355; (**B**) CID-MS^3 mass spectrum of the product ion at *m*/*z* 193.

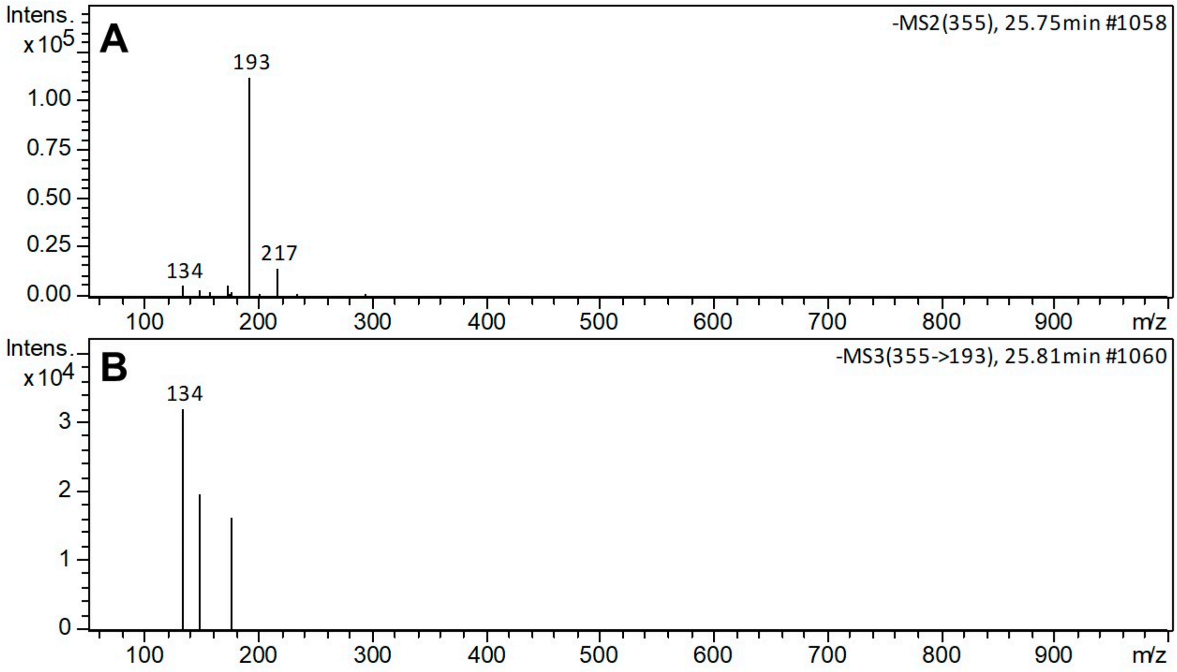

Figure S12. CID-MS^n mass spectra in negative ion mode for compound **12**: (**A**) CID-MS^2 spectrum of the precursor ion at *m*/*z* 355; (**B**) CID-MS^3 mass spectrum of the product ion at *m*/*z* 193.

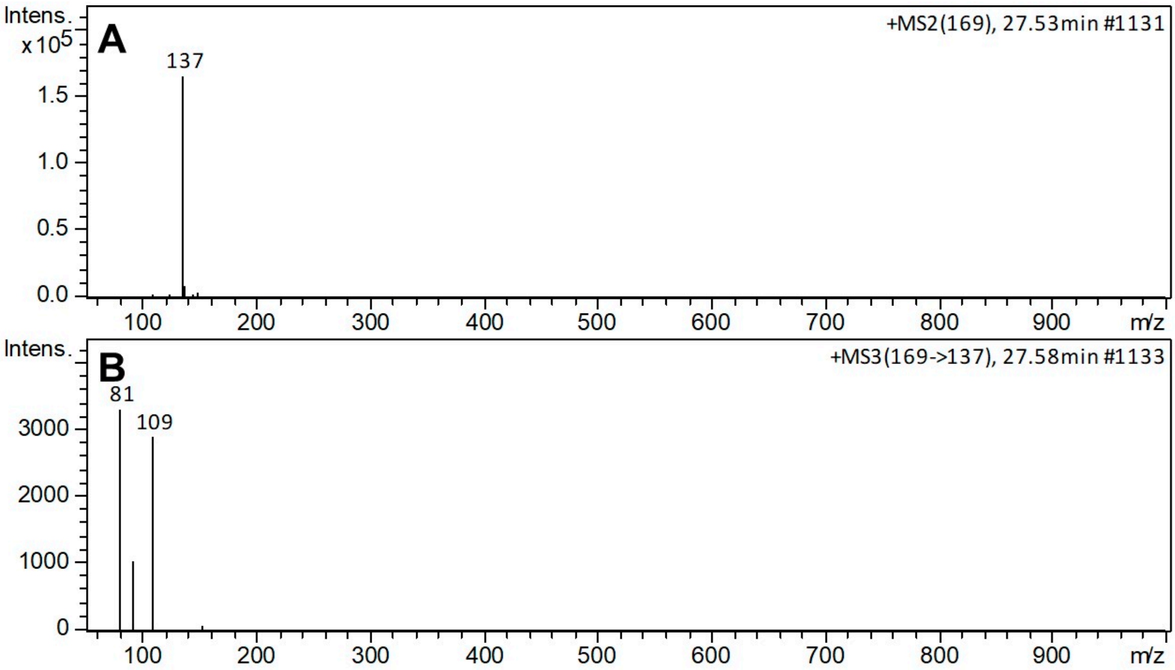

Figure S13. CID-MS^n mass spectra in positive ion mode for compound **13**: (**A**) CID-MS^2 spectrum of the precursor ion at *m*/*z* 169; (**B**) CID-MS^3 mass spectrum of the product ion at *m*/*z* 137.

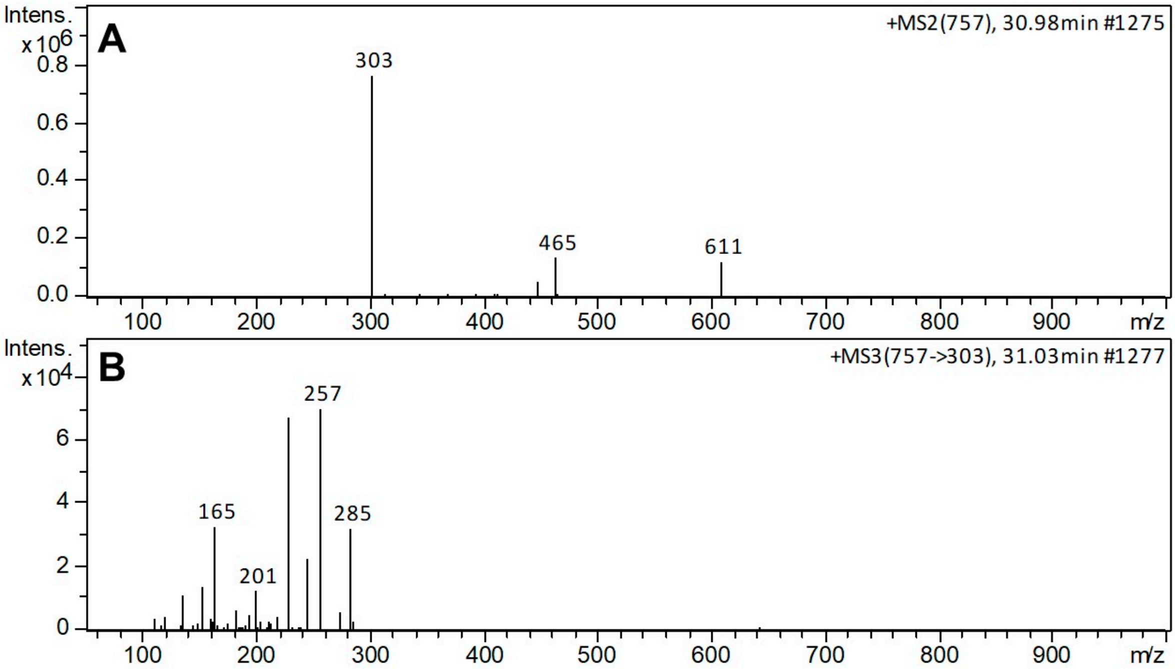

Figure S14. CID-MS^n mass spectra in positive ion mode for compound **14**: (**A**) CID-MS^2 spectrum of the precursor ion at *m*/*z* 757; (**B**) CID-MS^3 mass spectrum of the product ion at *m*/*z* 303.

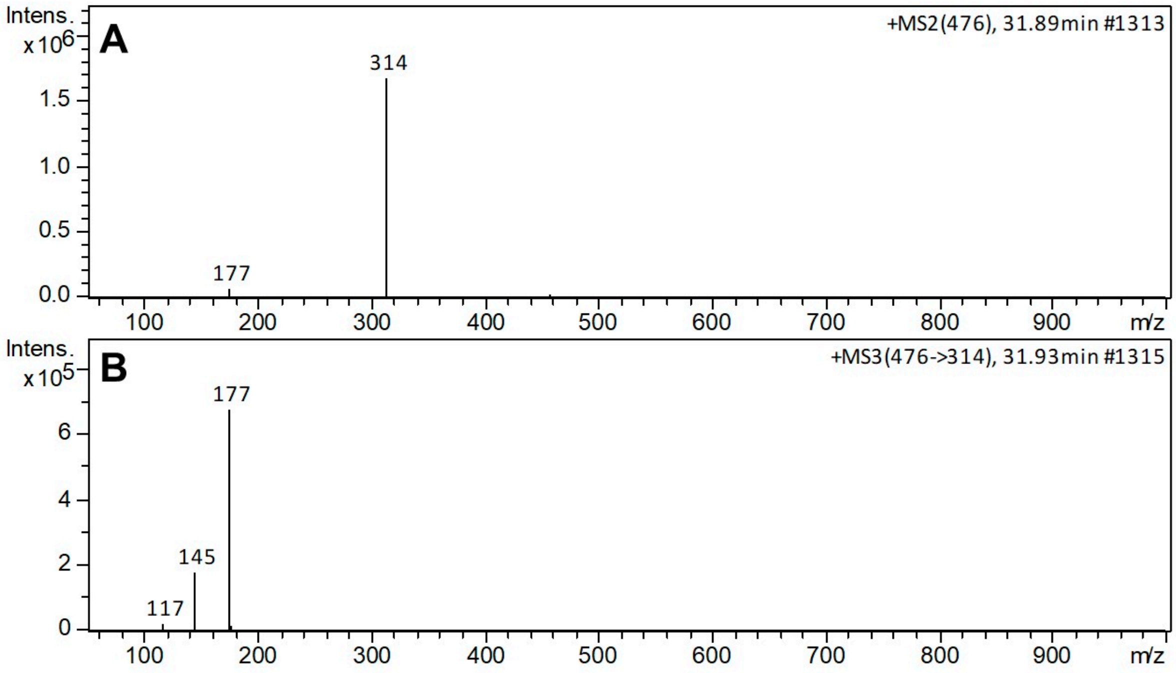

Figure S15. CID-MS^n mass spectra in positive ion mode for compound **15**: (**A**) CID-MS^2 spectrum of the precursor ion at *m*/*z* 476; (**B**) CID-MS^3 mass spectrum of the product ion at *m*/*z* 177.

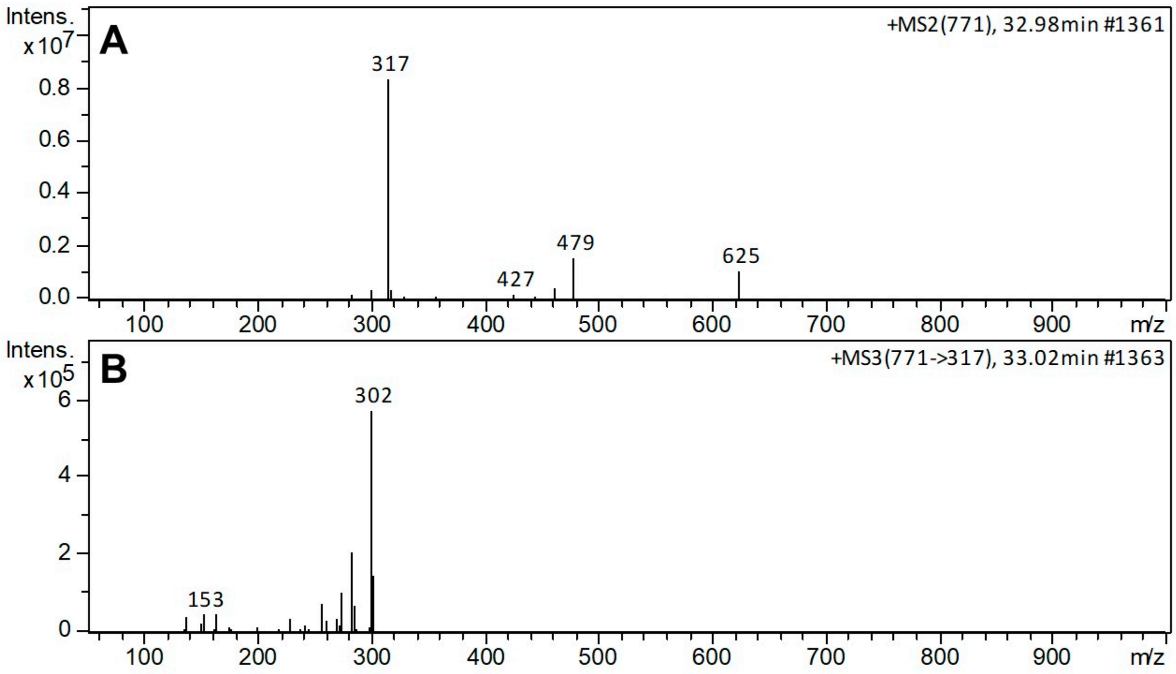

Figure S16. CID-MS^n mass spectra in positive ion mode for compound **16**: (**A**) CID-MS^2 spectrum of the precursor ion at *m*/*z* 771; (**B**) CID-MS^3 mass spectrum of the product ion at *m*/*z* 317.

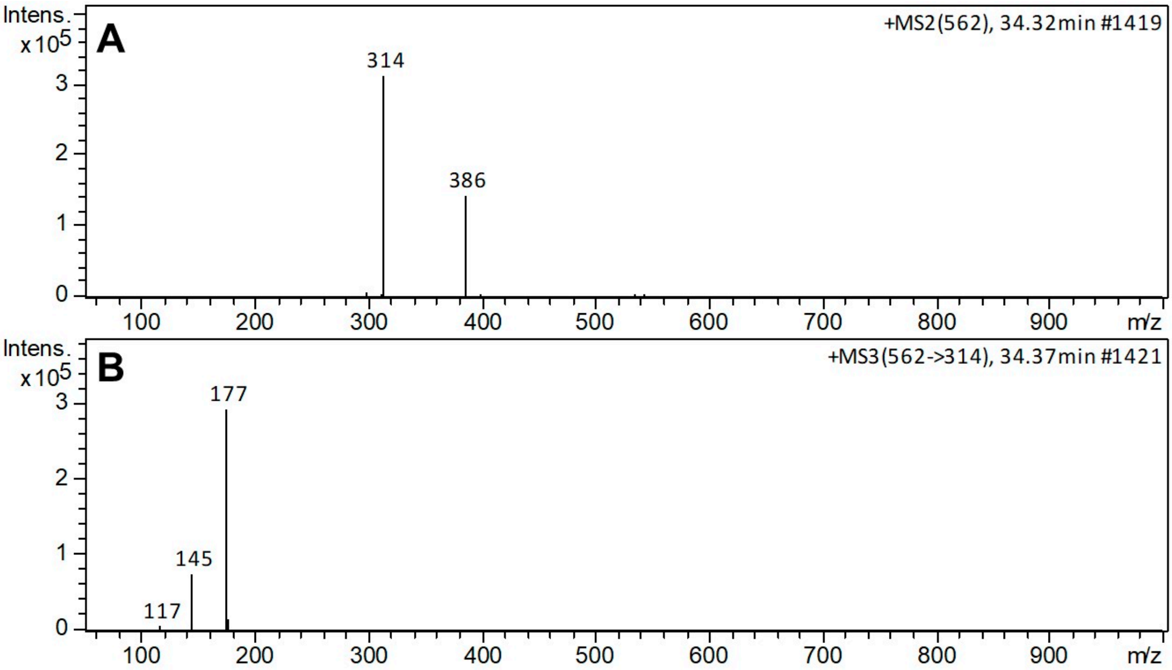

Figure S17. CID-MS^n mass spectra in positive ion mode for compound **17**: (**A**) CID-MS^2 spectrum of the precursor ion at *m*/*z* 562; (**B**) CID-MS^3 mass spectrum of the product ion at *m*/*z* 314.

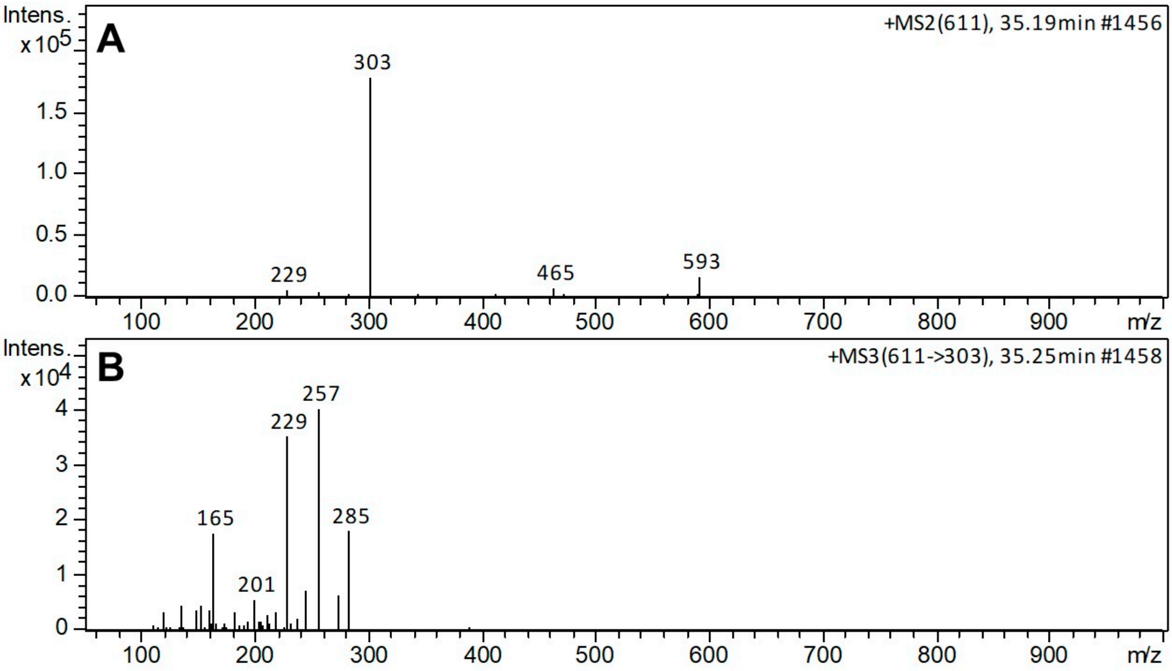

Figure S18. CID-MS^n mass spectra in positive ion mode for compound **18**: (**A**) CID-MS^2 spectrum of the precursor ion at *m*/*z* 611; (**B**) CID-MS^3 mass spectrum of the product ion at *m*/*z* 303.

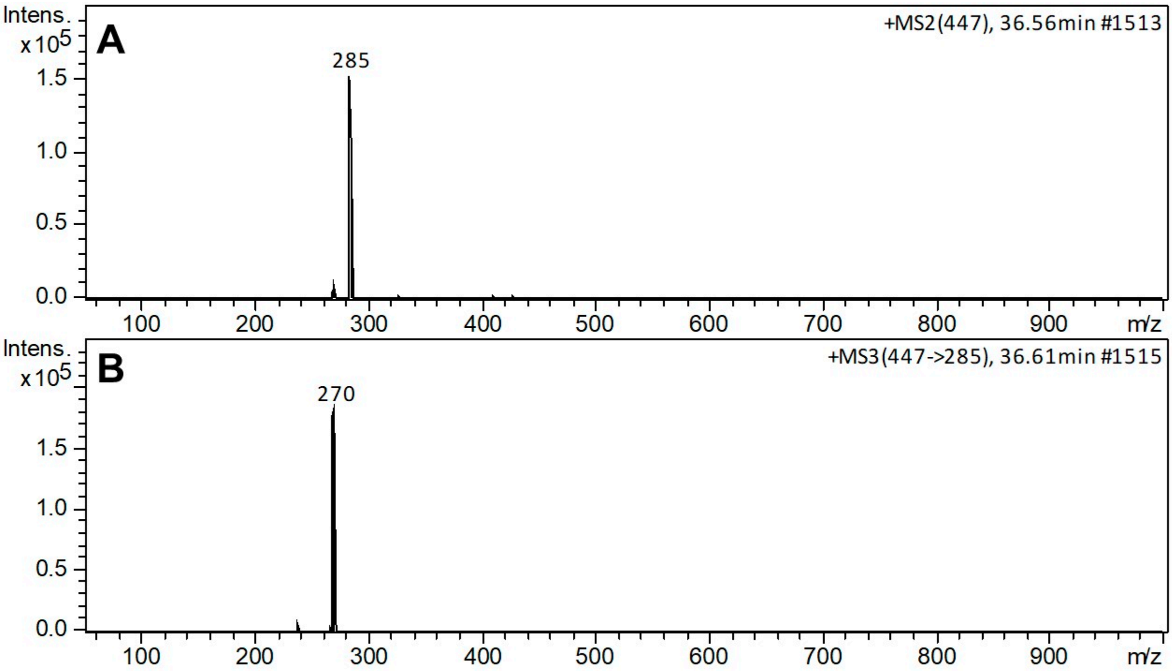

Figure S19. CID-MS^n mass spectra in positive ion mode for compound **19**: (**A**) CID-MS^2 spectrum of the precursor ion at *m*/*z* 447; (**B**) CID-MS^3 mass spectrum of the product ion at *m*/*z* 285.

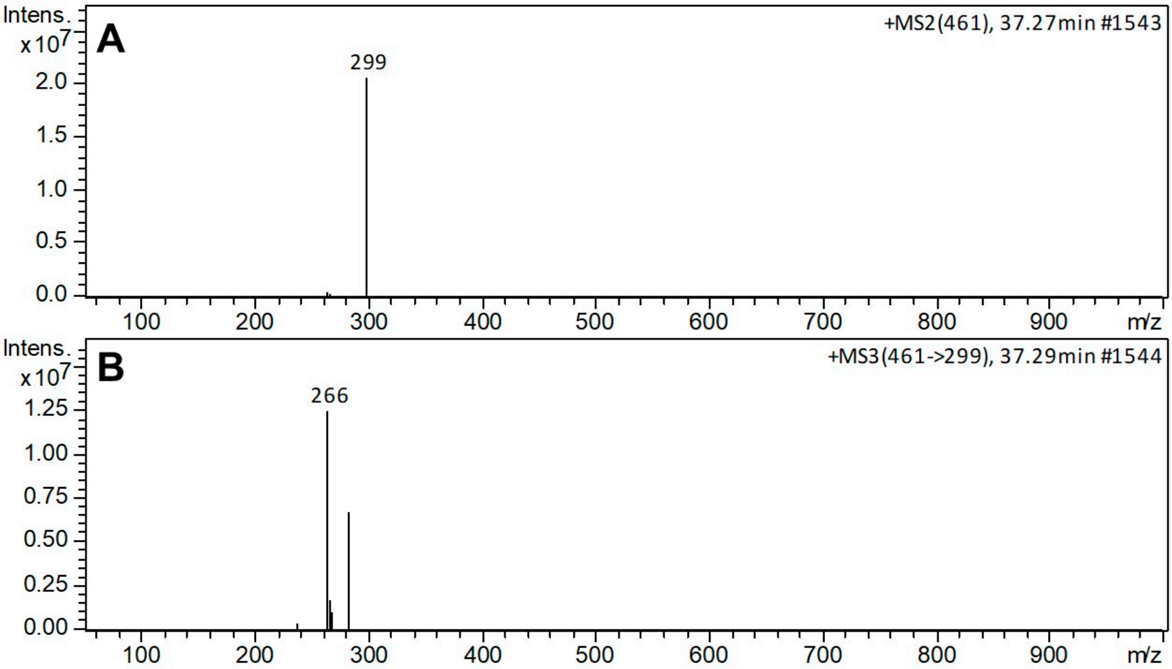

Figure S20. CID-MS^n mass spectra in positive ion mode for compound **20**: (**A**) CID-MS^2 spectrum of the precursor ion at *m*/*z* 461; (**B**) CID-MS^3 mass spectrum of the product ion at *m*/*z* 299.

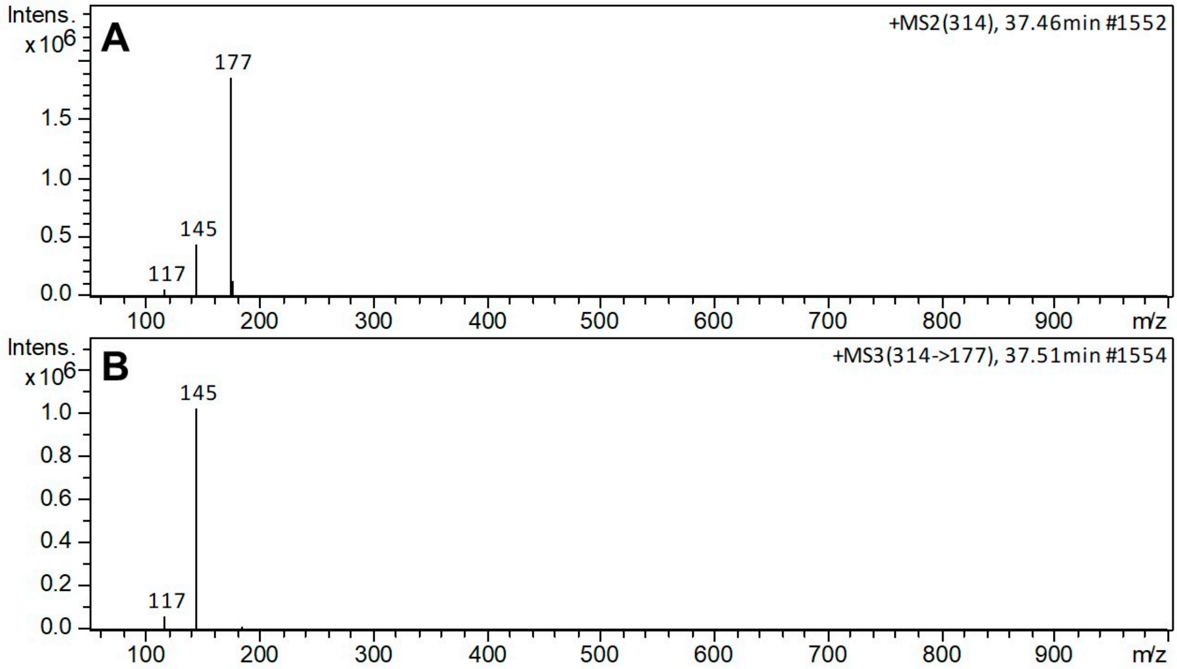

Figure S21. CID-MS^n mass spectra in positive ion mode for compound **21**: (**A**) CID-MS^2 spectrum of the precursor ion at *m/z* 314; (**B**) CID-MS^3 mass spectrum of the product ion at *m/z* 177.

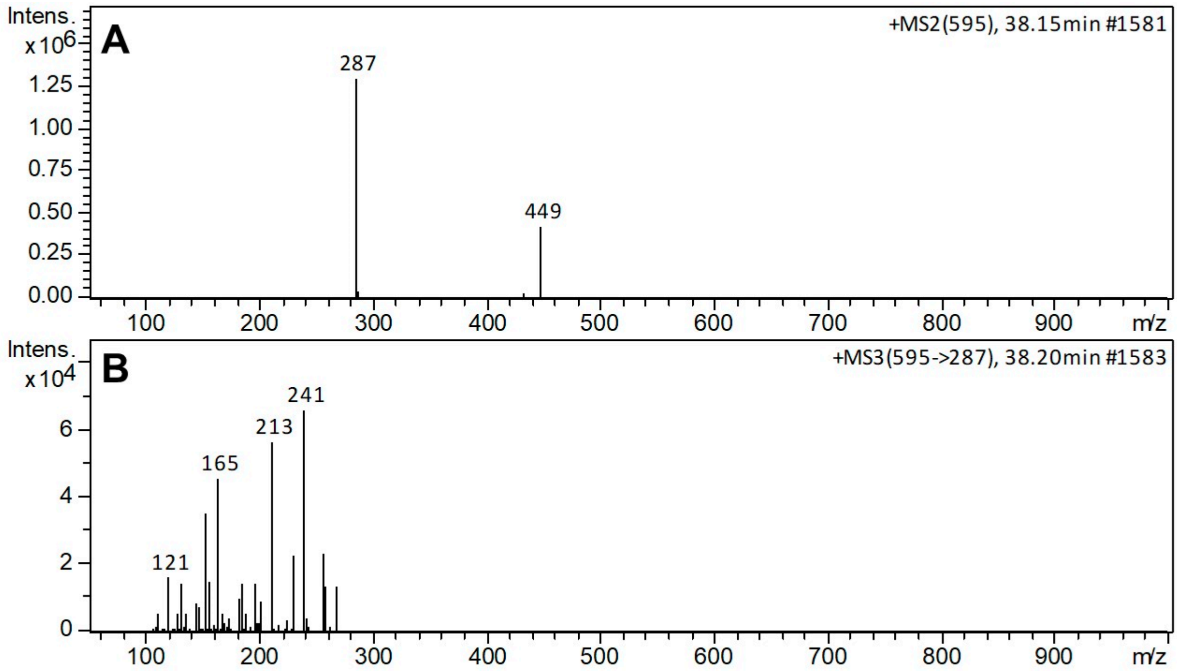

Figure S22. CID-MS^n mass spectra in positive ion mode for compound **22**: (**A**) CID-MS^2 spectrum of the precursor ion at *m/z* 595; (**B**) CID-MS^3 mass spectrum of the product ion at *m/z* 287.

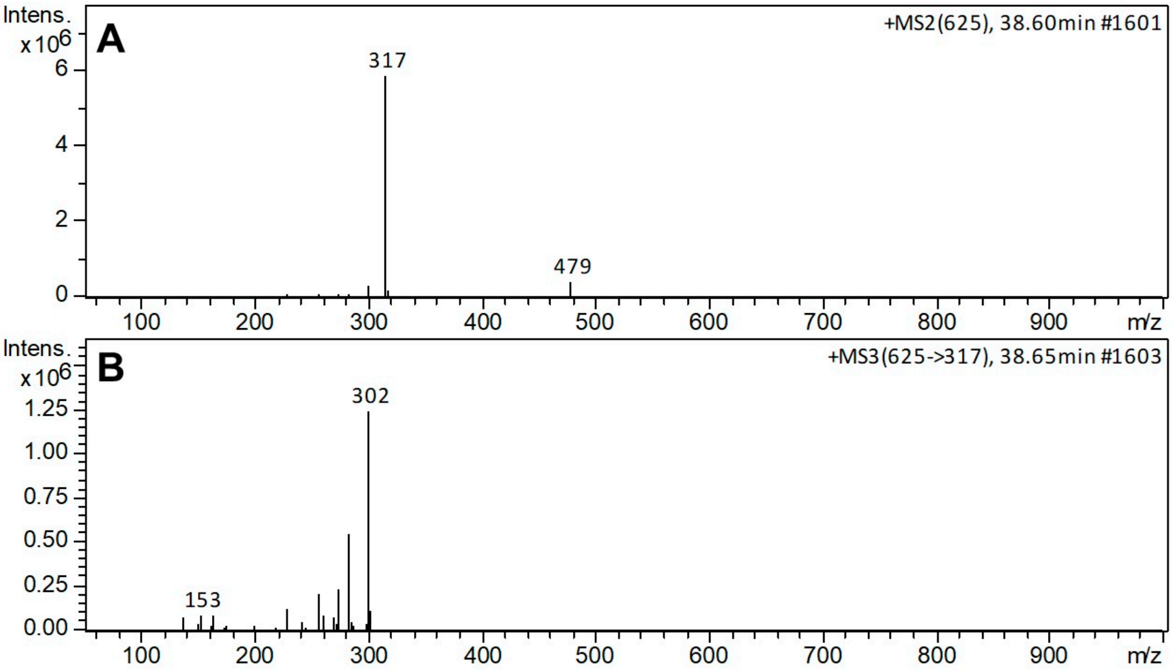

Figure S23. CID-MS^n mass spectra in positive ion mode for compound **23**: (**A**) CID-MS^2 spectrum of the precursor ion at *m/z* 625; (**B**) CID-MS^3 mass spectrum of the product ion at *m/z* 317.

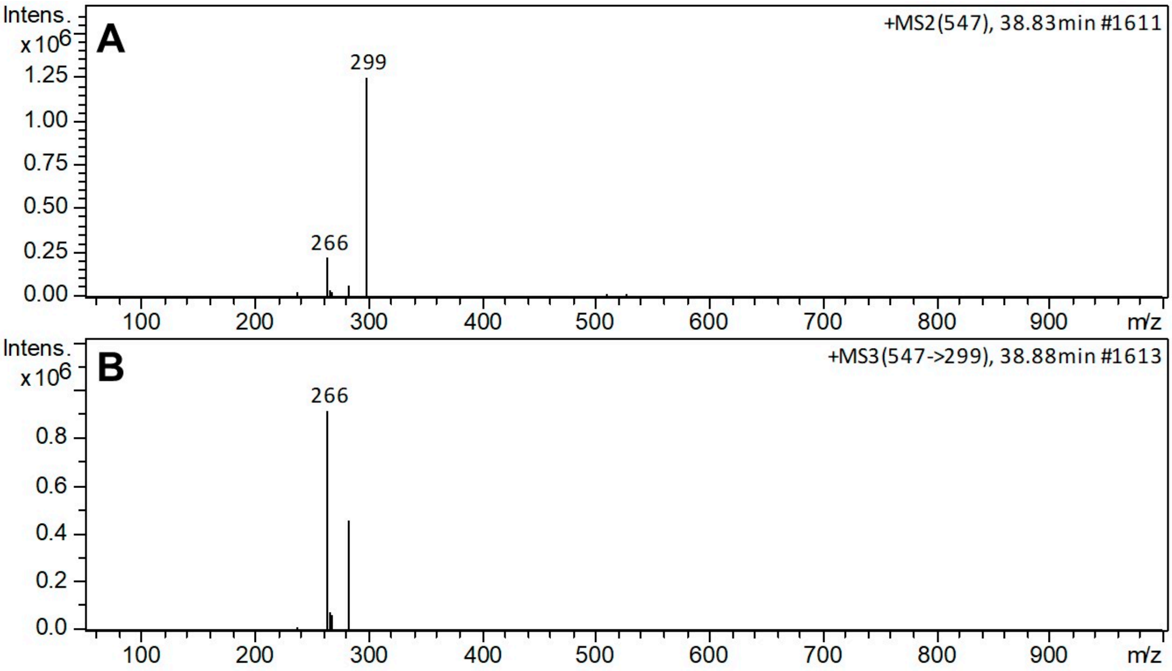

Figure S24. CID-MS^n mass spectra in positive ion mode for compound **24**: (**A**) CID-MS^2 spectrum of the precursor ion at *m/z* 547; (**B**) CID-MS^3 mass spectrum of the product ion at *m/z* 299.

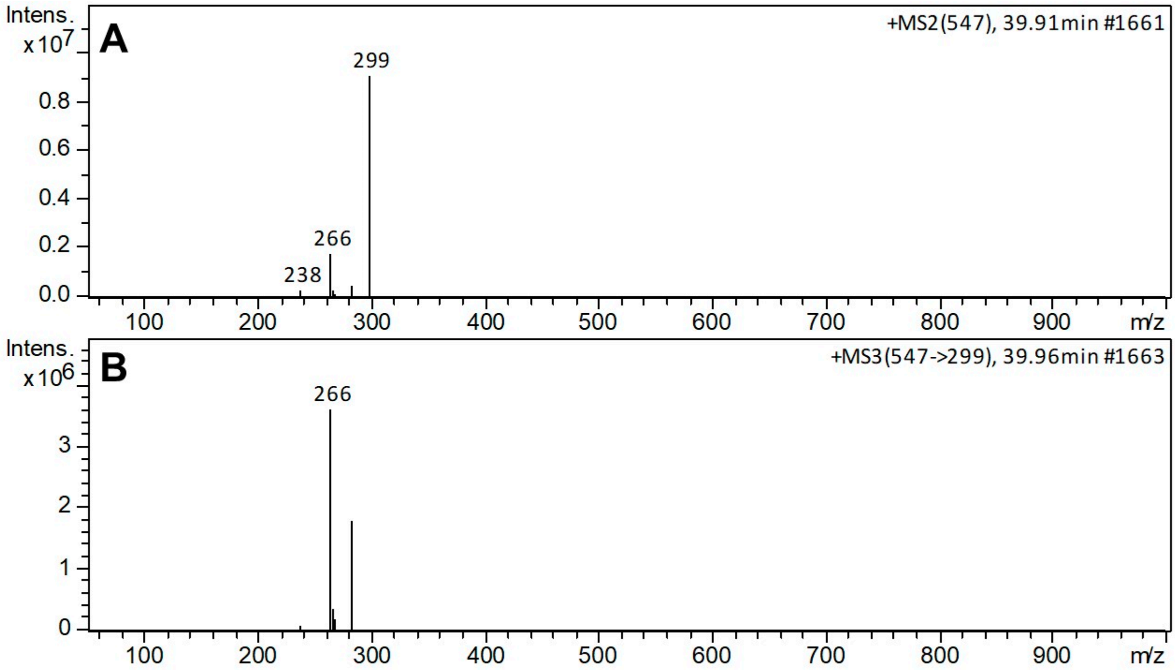

Figure S25. CID-MS^n mass spectra in positive ion mode for compound **25**: (**A**) CID-MS^2 spectrum of the precursor ion at *m*/*z* 547; (**B**) CID-MS^3 mass spectrum of the product ion at *m*/*z* 299.
